# C1q/tumor necrosis factor-related protein-3 (CTRP3) activated by forkhead box O4 (FOXO4) down-regulation protects retinal pericytes against high glucose-induced oxidative damage through nuclear factor erythroid 2-related factor 2 (Nrf2)/Nuclear factor-kappaB (NF-κB) signaling

**DOI:** 10.1080/21655979.2022.2031413

**Published:** 2022-02-23

**Authors:** XiuYa Zeng, YouYuan Peng, YanFeng Wang, KeMing Kang

**Affiliations:** a Department of Clinical Laboratory, The First Affiliated Hospital of Xiamen University, Xiamen, Fujian, China; b Xiamen Key Laboratory of Genetic Testing, Xiamen, China; c Department of Hepatobiliary Surgery, Zhongshan Hospital of Xiamen University, Xiamen, China; d Department of Ophthalmic Fundus Disease, Xiamen Eye Center of Xiamen University, Xiamen, China

**Keywords:** Human retinal pericytes, oxidative stress, CTRP3, FOXO4, nrf2/NF-κB signaling

## Abstract

Diabetic retinopathy (DR) remains a major cause of blindness among diabetes mellitus patients. C1q/tumor necrosis factor-related protein-3 (CTRP3) is a novel adipokine which is associated with multiple types of metabolism. Nevertheless, little is known about the role of CTRP3 in high glucose (HG)-induced human retinal pericytes (HRPs). This study set out to assess the influence of CTRP3 on HG-induced HRPs and elucidate the latent regulatory mechanism. RT-qPCR and Western blot were to analyze CTRP3 and forkhead box O4 (FOXO4) expression. Western blot was also utilized to detect the protein levels of apoptosis-related factors and nuclear factor erythroid 2-related factor 2 (Nrf2)/Nuclear factor-kappaB (NF-κB) signaling-related factors. CCK-8 was to measure cell proliferation while TUNEL assay was to estimate cell apoptosis. Levels of oxidative stress biomarkers including manganese (MnSOD), catalase (CAT) and malonedialdehyde (MDA) were evaluated by the corresponding kits. JASPAR database, ChIP and luciferase reporter assay were to verify the interaction between FOXO4 and CTRP3 promoter. The experimental results uncovered that CTRP3 expression was decreased in HG-stimulated HRPs. Moreover, CTRP3 overexpression strengthened the viability while abrogated the apoptosis and oxidative stress of HG-induced HRPs. Furthermore. FOXO4 was up-regulated in HG-induced HRPs. Besides, FOXO4 bond to CTRP3 promoter and inhibited CTRP3 transcription to modulate the Nrf2/NF-κB signaling pathway. FOXO4 up-regulation reversed the influence of CTRP3 elevation on the proliferation, apoptosis and oxidative stress of HG-induced HRPs. To be summarized, CTRP3 negatively modulated by FOXO4 prevented HG-induced oxidative damage in DR via modulation of Nrf2/NF-κB signaling.

## Graphical abstract

**Figure:**
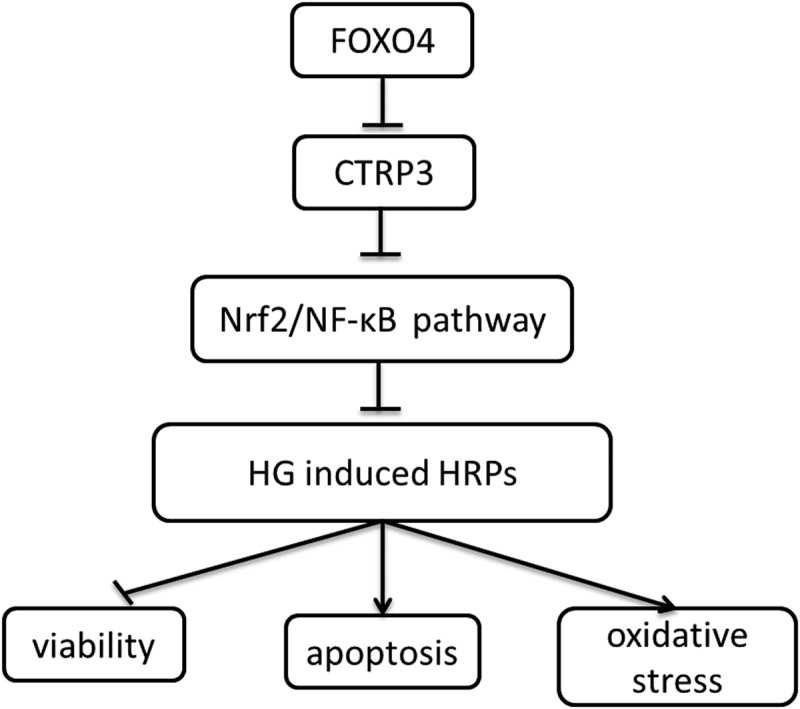

## Introduction

It is widely identified that diabetic retinopathy (DR) is a kind of ocular disease due to retina damage induced by diabetes [1]. As a frequent and specific microvascular complication of diabetes, DR is deemed as a leading cause of irreversible blindness in working-age adults [2]. Long diabetes duration, high blood pressure, poor metabolic control, sleep apnea syndrome are considered to be main risk factors for DR [3]. According to the World Health Organization, there will be more than 190 million diabetics worldwide by 2030. Among them, the incidence of DR in patients with diabetes over 20 years is 80%. At present, the clinical treatment of DR is mainly drug therapy, hormone therapy and surgical treatment [4]. However, very little is currently known about the pathogenesis of DR. Pericytes are vascular mural cells which regulate multiple pathological processes including angiogenesis, vascular remodeling, wound healing [5]. Unlike pericytes in many other organs, retinal pericytes (RPCs) play a critical role in the neurovascular unit [6]. More importantly, the dysfunction or loss of RPCs is one of the primary features of early stage of DR [7]. Hence, it is essential to explore the altered functions of RPCs to get a better understanding of the pathogenesis of DR.

CTRP3, an important member of CTRP family, is one kind of adipokine with a large quantity of biological effects including inflammation, angiogenesis, glucose and lipid metabolism [8]. Numerous reports have manifested that CTRP3 is determined as a crucial participator in cerebral ischemic stroke [9], severe acute pancreatitis [10], gestational diabetes mellitus [11] and so on. In the literature, CTRP3 has been clarified to mitigate the oxidative stress and apoptosis of retinal pigment epithelial cells under HG conditions [12]. Also, Yan et al. have proposed that serum CTRP3 level is down-regulated in type 2 diabetes mellitus patients and suppresses VCAM-1 production stimulated by high glucose and high lipid [13]. However, the specific regulatory mechanism of CTRP3 in DR has not been reported so far.

FOXO4, a member of human Forkhead-box (FOX) gene family, is known as a common transcription factor and implicated in the regulation of metabolism, oxidative stress resistance, cell proliferation and apoptosis [14]. It is well documented that FOXO4 plays the promoting role in myocardial ischemia-reperfusion injury [15] and acts as a suppressor in colorectal cancer [16], gastric cancer [17] and Cholangiocarcinoma [18]. However, whether FOXO4 interplays with CTRP3 in DR needs more exploration.

Nrf2/NF-κB signaling represents a functional cross-talk between two key transcription factors Nrf2 and NF-κB [19]. In addition, Nrf2/NF-κB signaling has been validated to exert influence on HG-induced oxidative stress and apoptosis of PRCs [20–22]. Thus Nrf2/NF-κB signaling is emerged as a key player in the pathogenesis of DR.

According to the above content, it is reasonable to assume that CTRP3 is regulated by FOXO4, thus regulating the oxidative stress damage of RPCs in DR. High glucose (HG) has been demonstrated to impair pericyte proliferation and induce pericyte apoptosis [23]. Therefore, HG was utilized to establish DR model in HRPs in this study. This study is designed to uncover the biological role of CTRP3 in the dysfunction of HRPs under HG conditions and illuminate the relationship among CTRP3, FOXO4 and Nrf2/NF-κB signaling.

## Materials and methods

### Cell culture

Primary HRPs (ACBRI 183) obtained from Cell Systems Corporation (Kirkland, WA, USA) were inoculated in Dulbecco’s modified Eagle’s medium (DMEM; Gibco). The medium was supplemented with 10% fetal bovine serum (FBS; Gibco, 11,573,397) and 1% antibiotics (streptomycin-penicillin) under the condition of 5% CO_2_ and 37°C.

To explore the damage to HRPs in DR, we constructed an in vitro DR model. HRPs were respectively maintained in 5.6 mmol/L of glucose as normal control (Control group), 5.6 mmol/L of glucose and 24.6 mmol/L of mannitol as osmotic pressure control (MA group), 30 mmol/L of glucose as high glucose group (HG group) [24].

## Cell transfection

Sangon Biotech Co., Ltd. (Shanghai, China) synthesized the overexpression lentivirus plasmids carrying CTRP3 gene (Ov-CTRP3) or FOXO4 gene (Ov-FOXO4) as well as the overexpression empty vectors (Ov-NC). Stable transfection cells were selected with puromycin for 2 weeks. Above plasmids were transfected into cells for gene overexpression for another 48 h using Lipofectamine 2000 (Invitrogen, Waltham, USA) in line with the manufacturer’s protocols.

## Reverse transcription-quantitative PCR (RT-qPCR)

With the adoption of RNA Extraction Kit (Omega Bio-tek Inc, Norcross, GA, U.S.A.), total RNA was extracted from indicated cells, followed by reverse transcription into cDNA by Primescript™ RT reagent kit (Takara, Dalian, China). PCR reactions were prepared through SYBRGreen Master Mix (Bio-Rad, USA) on the 7500HT Fast Real Time PCR system (Applied Biosystems, Foster City, CA, USA) with GAPDH as the endogenous reference. The primer sequence were as follows: CTRP3, forward, 5’-ATGCTTTGGAGGCAGCTCAT-3’, reverse, 5’-TCACCTTTGTCGCCCTTCTC-3’; FOXO4, forward, 5’-CCAGAGATCGCTAACCAGCC-3’, reverse, 5’-TTTCAATGGCCTTTTCCCCCA-3’. Relative mRNA levels of CTRP3 and FOXO4 were measured with the adoption of 2^−ΔΔCt^ methods [25].

## Cell Counting Kit-8 (CCK-8) assay

HRPs were inoculated into 96-well plates at a density of 5,000 cells per well and incubated at 37°C. After indicated treatment, 10 μl CCK-8 solution (40203ES60, Yeasen Biotechnology, Shanghai, China) was added to each well. After being maintained at 37°C for 2 h, the measurement of absorbance at 450 nm was carried out with a microplate reader (Bio-Tek Technologies, Winooski, VT, United States).

## Terminal-deoxynucleotidyl Transferase Mediated Nick End Labeling (TUNEL) assay

TUNEL assay was performed by the in situ cell death detection kit (Roche) in compliance with the manufacturer’s instructions. In short, HRPs were fixed using 4% paraformaldehyde (Sigma-Aldrich), after which cells were washed with PBS and 0.1% Triton X-100 was utilized to permeabilize these washed cells. DAPI was used to label the nuclei staining for 10 min. Finally, the apoptotic cells were observed under a fluorescence microscope (Nikon Instruments Inc., Melville, NY, USA).

## Detection of MnSOD, CAT and MDA

Cells were decomposed in 300 μl lysis buffer and total protein was quantified by BCA kit (Bio-Rad Laboratories, Inc., Hercules, CA, USA). A commercially available Ransod kit (Randox, Laboratories Ltd. Ardmore, UK), Catalytic enzymes activity kit and MDA assay kit from Nanjing Jiancheng Bioengineering Co. Ltd. were employed to incubate with total protein to respectively determine the MnSOD activity, CAT activity and MDA activity in accordance with the supplier’s guidance. The absorbance was determined at 450 nm using a microplate reader (Thermo, Minneapolis, MN, USA).

## Chromatin immunoprecipitation (ChIP)

A commercially available kit for DNA gel extraction (Beyotime, Shanghai, China) was utilized in the ChIP assay [26]. After cells were cross-linked with 1% formaldehyde, they were randomly fragmented by ultrasonic. Stained chromatin was incubated with FOXO4 antibody (Abcam, 1:1000, ab128908) or IgG antibody (Abcam, 1:1000, ab109489) as control in magnetic beads. The enrichment of precipitated chromatin DNA was subjected to PCR analysis.

## Luciferase reporter assay

After the construction of the wild type and mutant sequences of CTRP3 promoter region into the pGL3 vector (Promega Corporation, Madison, WI, USA), they were co-transfected along with Ov-FOXO4 plasmids or the empty vector into cells. After 48 h, the luciferase activity was subjected to the detection from Luciferase Reporter Assay System (Promega, Madison, WI) [27].

## Western blot

Protein samples collected in RIPA lysis buffer (Sigma-Aldrich; Merck KGaA) were added to 10% SDS-PAGE for electrophoresis and moved to PVDF membranes (Millipore, USA). The membranes were impeded by 5% nonfat milk and incubated with the following primary antibodies: anti-CTRP3 (Abcam, ab36870), anti-B cell lymphoma-2 (Bcl-2) (Abcam, 1:1000, ab32124), anti-BCL-2 associated X (Bax) (Abcam, 1:1000, ab32503), anti-cleaved caspase 3 (Abcam, 1:500, ab32042), anti-caspase 3 (Abcam, 1:5000, ab32351), anti-cleaved Poly (ADP-ribose) polymerase (PARP) (Abcam, 1:1000, ab32064), PARP (Abcam, 1:1000, ab191217), anti-FOXO4 (Abcam, 1:1000, ab128908), anti-Nrf2 (Abcam, 1:1000, ab62352), anti- phosphorylated (p-)NF-κB p65 (Abcam, 1:1000, ab76302), anti-NF-κB p65 (Abcam, 1:1000, ab32536) and anti-GAPDH (Abcam, 1:2500, ab9485) at 4°C overnight. Afterward, the membranes were incubated with HRP‐conjugated goat anti-rabbit IgG (Abcam, 1:1000, ab109489) at room temperature for 1 h. Then, the blots were detected by the ECL detection kit (Roche) and analyzed with the help of Image J software (NIH, Bethesda, MD, USA).

## Bioinformatics tools

With the application of JASPAR database (https://jaspar.genereg.net/), the possible binding sites between FOXO4 and CTRP3 promoter were predicted [28].

## Statistical analyses

Statistical analysis in this study were completed using GraphPad Prism 8.0 software (GraphPad Software, Inc.) with the employment of Student’s t-test and one-way analysis of variance (ANOVA) as well as Tukey’s post hoc test. All experimental data were exhibited as the mean ± SD and the data was determined to be statistically significant when p < 0.05.

## Results

### Overexpression of CTRP3 enhances the viability of HG-induced HRPs

**Figure 1.**
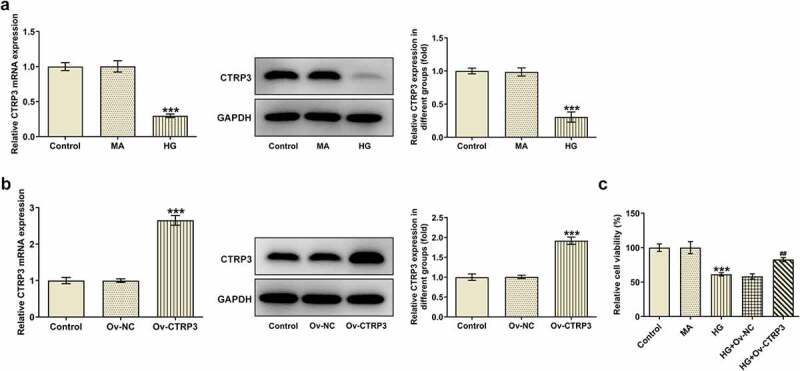
Overexpression of CTRP3 enhances the viability of HG-induced HRPs. (a) CTRP3 expression in control group, MA group and HG group was examined through RT-qPCR and Western blot. ***P < 0.001 vs. MA. (b) The transfection efficiency of Ov-CTRP3 plasmid was tested by RT-qPCR and Western blot. ***P < 0.001 vs. Ov-NC. (c) Cell proliferation was evaluated by CCK-8 assay. ***P < 0.001 vs. MA. ^##^P < 0.01 vs. HG+Ov-NC. CTRP3, C1q/tumor necrosis factor-related protein-3. HG, high glucose. MA, mannitol.

First of all, CTRP3 expression was detected with the employment of RT-qPCR and Western blot analysis. The results indicated that after induced by HG, CTRP3 expression was discovered to be decreased in HRPs in comparison with the MA group (Figure 1(a)). For the subsequent experiments, CTRP3 expression was enhanced by transfection of Ov-CTRP3 plasmid and the transfection efficiency was tested by RT-qPCR and Western blot analysis (Figure 1(b)). As demonstrated in Figure 1c, the results from CCK-8 assay revealed that HRPs viability was attenuated under HG conditions while this effect was restored by up-regulation of CTRP3. In a word, CTRP3 prevented the loss of cell viability of HRPs under HG conditions.

### CTRP3 hampers HG-stimulated HRPs apoptosis

**Figure 2.**
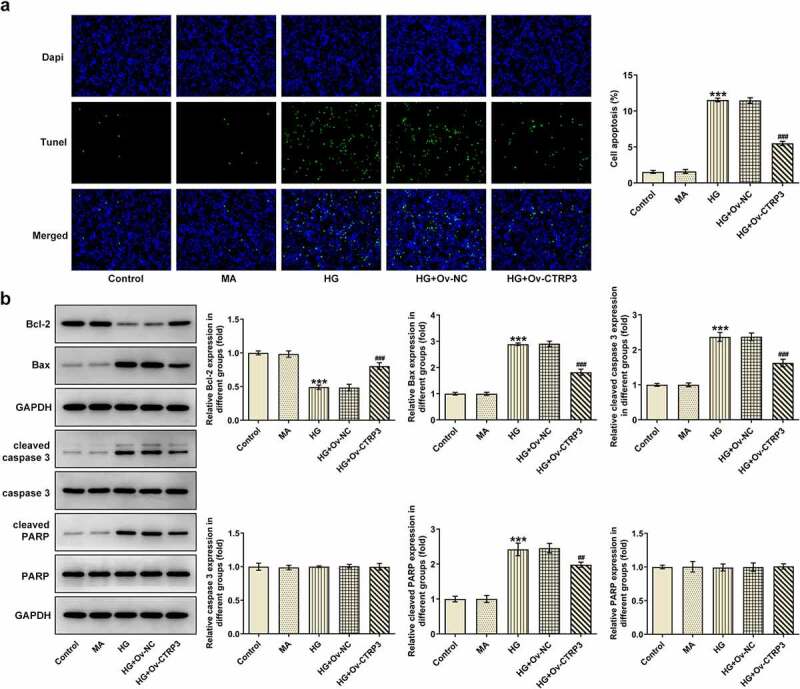
CTRP3 hampers HG-stimulated HRPs apoptosis. (a) Cell apoptosis was appraised by TUNEL assay. (b) The protein levels of apoptosis-related factors were analyzed by Western blot. ***P < 0.001 vs. MA. ^##^P < 0.01, ^###^P < 0.001 vs. HG+Ov-NC. CTRP3, C1q/tumor necrosis factor-related protein-3. HG, high glucose. Bcl-2, B cell lymphoma-2. Bax, BCL-2 associated X. cleaved PARP, cleaved Poly (ADP-ribose) polymerase. PARP, Poly (ADP-ribose) polymerase.

To detect the effect of CTRP3 on HRPs apoptosis induced by HG, the apoptotic ability of HRPs was assessed by TUNEL assay and Western blot analysis. From TUNEL assay, it was clearly observed that HG-induced HRPs apoptosis was rescued by CTRP3 elevation (Figure 2(a)). Similarly, the protein levels of apoptosis-related factors including Bcl-2, Bax, cleaved caspase 3, caspase 3, cleaved PARP and PARP were analyzed by Western blot. It was observed that the decrease in Bcl-2 protein level and the increase in Bax, cleaved caspase 3, cleaved PARP protein levels triggered by HG exposure were all offset by up-regulation of CTRP3 while no apparent changes were noticed in the protein levels of caspase 3 and PARP (Figure 2(b)). In short, CTRP3 played the suppressive role in the apoptosis of HRPs exposed to HG.

### CTRP3 ameliorates HG-mediated oxidative stress of HRPs

**Figure 3.**
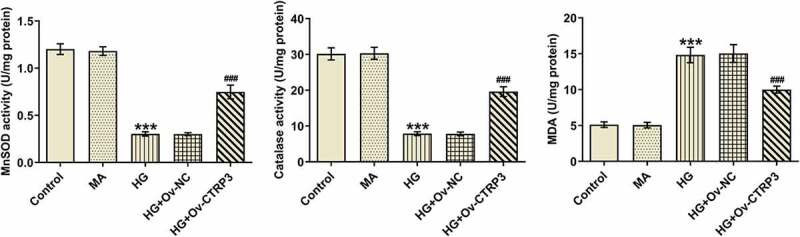
CTRP3 ameliorates HG-mediated oxidative stress of HRPs. Activities of MnSOD, CAT and MDA were measured by kits. ***P < 0.001 vs. MA. ^###^P < 0.001 vs. HG+Ov-NC. CTRP3, C1q/tumor necrosis factor-related protein-3. HG, high glucose. MA, mannitol. MnSOD, manganese. CAT, catalase. MDA, malonedialdehyde.

Oxidative stress is a key driver in the progression of DR [29]. Thereafter, the levels of oxidative stress markers including MnSOD, CAT and MDA were examined by the corresponding kits. As a result, HG treatment improved MDA level and lessened MnSOD and CAT levels, while this result was countervailed after CTRP3 was overexpressed (Figure 3). The results reveled that CTRP3 ameliorates HG-mediated oxidative stress of HRPs.

### Transcription factor FOXO4 inhibits the transcription of CTRP3

**Figure 4.**
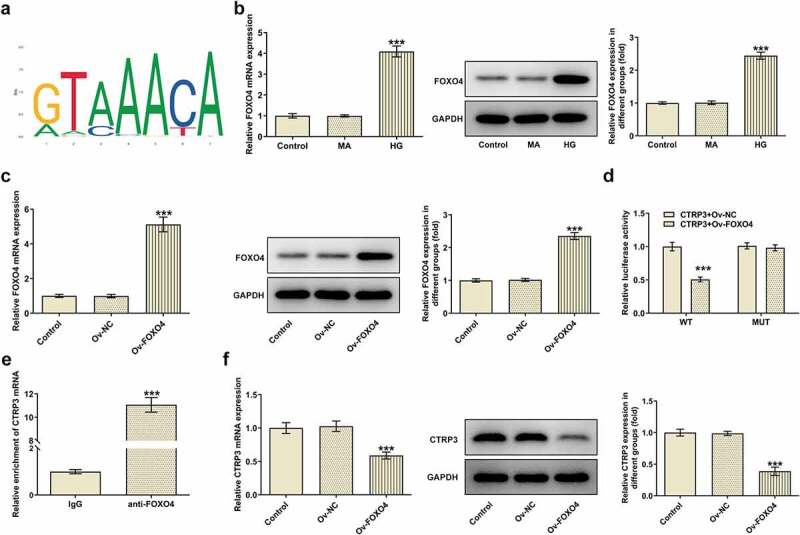
Transcription factor FOXO4 inhibits the transcription of CTRP3. (a) Binding motif of FOXO4 with CTRP4 promoter was predicted by JASPAR database. (b) FOXO4 expression in Control group, MA group and HG group was examined through RT-qPCR and Western blot. ***P < 0.001 vs. MA. (c) The transfection efficiency of Ov-FOXO4 plasmid was tested by RT-qPCR and Western blot. ***P < 0.001 vs. Ov-NC. (d) The luciferase activities of CTRP3 WT promoter and CTRP3 MUT promoter after transfection of Ov-FOXO4 were detected by luciferase reporter assay. (e) RIP assay confirmed the abundance of CTRP3 in FOXO4 antibody. ***P < 0.001 vs. IgG. (f) CTRP3 expression in Control group, Ov-NC group and Ov-FOXO4 group was examined through RT-qPCR and Western blot. ***P < 0.001 vs. Ov-NC. CTRP3, C1q/tumor necrosis factor-related protein-3. HG, high glucose. MA, mannitol. FOXO4, forkhead box O4.

Subsequently, we further explored the regulation mechanism of CTRP3 on HG-induced HRPs in DR. Intriguingly, with the employment of JASPAR database, the potent binding site between FOXO4 and CTRP3 promoter was predicted and displayed in Figure 4a. Moreover, RT-qPCR and Western blot detected elevated FOXO4 expression at mRNA and protein level in HRPs after HG treatment relative to the MA group (Figure 4(b)). Subsequently, HG-induced HRPs were transfected with Ov-FOXO4 to successfully up-regulate FOXO4 expression and the transfection efficiency was tested by RT-qPCR and Western blot (Figure 4(c)). Meanwhile, luciferase reporter assay verified that elevation of FOXO4 dramatically decreased the luciferase activity of CTRP3 promoter (Figure 4(d)), which implied the possible interaction between FOXO4 and CTRP3 promoter. Similarly, ChIP assay confirmed that CTRP3 was enriched in FOXO4 antibody (Figure 4e), suggesting the strong affinity of FOXO4 with CTRP4 promoter. Eventually, it turned out that overexpression of FOXO4 cut down the mRNA and protein level of CTRP3 (Figure 4f). To sum up, FOXO4 functioned as a transcription inactivator of CTRP3.

### CTRP3 inactivated by FOXO4 modulates the Nrf2/NF-κB signaling pathway

**Figure 5.**
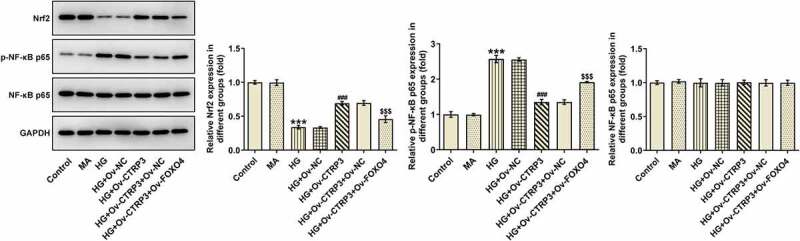
CTRP3 inactivated by FOXO4 modulates the Nrf2/NF-κB signaling pathway. The protein levels of Nrf2, p-NF-κB p65 and NF-κB p65 were analyzed by Western blot. ***P < 0.001 vs. MA. ^###^P < 0.001 vs. HG+Ov-NC. ^$$$^P < 0.001 vs. HG+Ov-CTRP3+ Ov-NC. CTRP3, C1q/tumor necrosis factor-related protein-3. HG, high glucose. MA, mannitol. FOXO4, forkhead box O4. Nrf2, nuclear factor erythroid 2-related factor 2. p-NF-κB p65, phosphorylated-Nuclear factor-kappaB p65. NF-κB p65, Nuclear factor-kappaB p65.

Further, the protein levels of Nrf2, p-NF-κB p65 and t-NF-κB p65 were analyzed with the application of Western blot. As a result, HG treatment decreased Nrf2 protein level and elevated p-NF-κB p65 protein level. Under this condition, overexpression of CTRP3 increased Nrf2 protein level and reduced p-NF-κB p65 protein level, whereas this effect was restored again by up-regulation of FOXO4 (Figure 5). Collectively, overexpression of FOXO4 inhibited the regulatory role of CTRP3 in HG-induced Nrf2 signaling inactivation and NF-κB signaling activation.

### CTRP3 mediated by FOXO4 regulates HRPs viability, apoptosis and oxidative stress under HG conditions through Nrf2/NF-κB signaling

**Figure 6.**
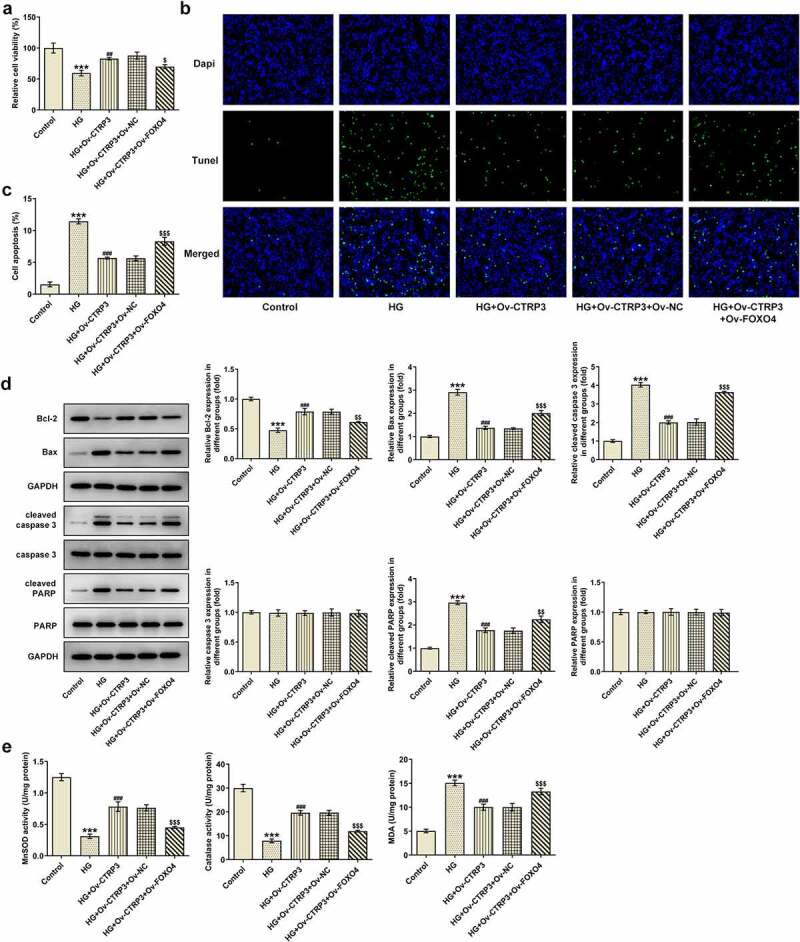
CTRP3 mediated by FOXO4 regulates HRPs viability, apoptosis and oxidative stress under HG conditions through Nrf2/NF-κB signaling. (a) Cell proliferation was evaluated by CCK-8 assay. (b) Cell apoptosis was appraised by TUNEL assay and (c) quantification. (d) The protein levels of apoptosis-related factors were analyzed by Western blot. (e) Activities of MnSOD, CAT and MDA were measured by kits. ***P < 0.001 vs. Control. ^##^P < 0.01, ^###^P < 0.001 vs. HG. ^$^P < 0.005, ^$$^P < 0.01, ^$$$^P < 0.001 vs. HG+Ov-CTRP3+ Ov-NC. CTRP3, C1q/tumor necrosis factor-related protein-3. HG, high glucose. FOXO4, forkhead box O4. Bcl-2, B cell lymphoma-2. Bax, BCL-2 associated X. cleaved PARP, cleaved Poly (ADP-ribose) polymerase. PARP, Poly (ADP-ribose) polymerase. MnSOD, manganese. CAT, catalase. MDA, malonedialdehyde.

For the purpose of the verification of the interaction between CTRP3 and FOXO4 in HG-induced HRPs, rescue assays were carried out. CCK-8 assay substantiated that CTRP3 exacerbated the inhibited HRPs proliferation caused by HG, while this result was counteracted by FOXO4 overexpression (Figure 6(a)). On the contrary, the protective role of CTRP3 against HG-induced HRPs apoptosis was repressed by overexpression of FOXO4 (Figure 6(b-c)). The similar result could also be seen in Western blot analysis, accompanied by the finding that the reduced protein level of Bcl-2 and the elevated protein levels of Bax, cleaved caspase 3 and cleaved PARP in HG-stimulated HRPs were all restored by CTRP3 up-regulation and reversed again by FOXO4 elevation (Figure 6(d)). Besides, the inhibitory effect of CTRP3 on HG-induced oxidative stress was impeded after FOXO4 was overexpressed, as evidenced by the result that FOXO4 cut down the enhanced MnSOD and CAT levels while augmented the decreased MDA level due to CTRP3 overexpression (Figure 6(e)). Collectively, the influence of CTRP3 on the viability, apoptosis and oxidative stress of HRPs upon exposure to HG was all abrogated by FOXO4 up-regulation.

## Discussion

Early and selective death of pericytes is considered to be one of the main features of DR pathogenesis [30]. What’s more, it is believed that pericytes are susceptible to the metabolic abnormalities, thereby contributing to the development of DR even diabetes mellitus [31]. The most important early manifestations of DR are the reduction or disappearance of HRPs, pathological capillary endothelial cell proliferation, basement membrane thickening, blood-retinal barrier function destruction, retinal ischemia and hypoxia, resulting in pathological neangiogenesis [32]. And late proliferative DR appears, which is also an important cause of blindness in diabetic patients. Therefore, we chose HRPs for experiment Oxidative stress is recognized as a metabolic condition derived from an imbalance between the production of oxygen radicals and their antioxidant capacity [33]. In detail, oxidative stress, a consequence of overproduction of reactive oxygen species (ROS) and the imbalance of the antioxidant defense systems for ROS elimination, plays a central role in a wide variety of biological processes of DR [29]. Moreover, during that process, the break of the balance leads to retinal cell injury for the reason that the retina is sensitive to ROS due to high-energy demands and light exposure [34]. As one of the antioxidant enzymes, MnSOD serves as a main defense against oxidative stress [35]. Also, CAT is a kind of natural antioxidase capable of scavenging ROS [36]. MDA is the most commonly measured indicator of oxidative stress [37]. Therefore, MnSOD, CAT and MDA activities were estimated for detection of the oxidative stress levels in HRPs in this study. HG condition mediating connexin expression and gap junction intercellular communication is implicated in the initiation and development of DR, a glucose-related disease [23]. Hence, HRPs were treated with HG to establish a DR model in this study and the experimental results disclosed that after stimulated by HG, the viability of HRPs was alleviated while the apoptosis and oxidative stress of HRPs were both exacerbated.

Current researches have certified that CTRP3 exerts an enormous function on human diseases, such as severe acute pancreatitis [10], cerebral ischemic stroke [9], gestational diabetes mellitus [11] and cancer [38]. A previous study has shown that CTRP3 reduces the proliferation and ECM production of glomerular mesangial cells induced byHG through inactivation of JAK2/STAT3 signaling pathway [39]. More importantly, Zhang et al. have elaborated that CTRP3 plays an inhibitory role in the apoptosis and oxidative stress of retinal pigment epithelial cells exposed to HG [12]. CTRP3 can protect human umbilical vein endothelial cell injury induced by high glucose [40]. Yan et al. have illustrated that CTRP3 exhibits low serum levels in type 2 diabetes mellitus patients and could be viewed as a novel hallmark for DR [13]. Consistent with these findings, CTRP3 was discovered to be lowly expressed in HRPs under HG conditions. Meanwhile, gain-of-function experiments testified that up-regulation of CTRP3 aggravated the viability of HG-stimulated HRPs while attenuated the apoptosis and oxidative stress of HG-induced HRPs.

Transcription factor FOXO4 has been regarded as a mediator of cellular gene regulation. For instance, Arg1 transcription is activated by FOXO4 in myocardial infarction [41]. FOXO4 suppresses the transcription of USP10 to boost acute myocardial infarction [42]. Functionally, it is also well documented that FOXO4 chiefly serves as a suppressor in different types of tumors [43]. At the same time, FOXO4 overexpression has been exposed to drive the process of DR, and its overexpression abrogates the antioxidant and anti-apoptotic effects of α-melanocyte-stimulating hormone in HG-stimulated retinal vascular endothelial cells [44,45]. It was noticed in our experiments that FOXO4 expression was increased in HG-stimulated HRPs. In addition, FOXO4 had a strong affinity with CTRP3 promoter and hindered the transcription of CTRP3. Rescue assays eventually verified that the enhanced viability, the inhibited apoptosis and oxidative stress of HG-mediated HRPs on account of overexpression of CTRP3 were all reversed by up-regulation of FOXO4.

The understanding of the regulatory mechanism of Nrf2 activity and its downstream signaling pathways have great significance in multiple cellular processes, such as inflammation, autophagy and metabolism [46]. Additionally, it turns out that Nrf2 plays a protective role against oxidative stress [20,47]. NF-κB is also a pivotal determinant in various biological processes and phosphorylation of IκBs could release NF-κB to result in nuclear translocation and activation of gene transcription [48]. The interplay between Nrf2 and NF-κB constitutes Nrf2/NF-κB signaling in which Nrf2 deficiency can augment NF-κB activity, whereas NF-κB can modulate Nrf2 transcription and activity [19]. The combination of the two is involved in the development of a variety of diseases. For example, protocatechuic acid impacts cerebral aneurysm through TNF-α/NF-κB/Nrf-2 signaling [49]. Egg yolk oils eases inflammatory response via Nrf2/NF-κB pathway [50]. Furthermore, NF-κB pathway modulates pericyte apoptosis in DR [21]. Activation of NF-κB stimulates retinal pericyte apoptosis [22]. As reported, CTRP3 is an activator in Nrf2 pathway in HG-induced retinal pigment epithelial cells [12]. More intriguingly, Lv et al. have demonstrated that CTRP3 decreases p-NF-κB p65 expression and p53 acetylation [10]. Sang et al. have validated that FOXO4 cuts down NF-κB and p-NF-κB p65 expressions and inactivates NF-κB signaling in alcohol-induced chronic liver injury [51]. The experimental results in this study confirmed that after stimulated by HG, Nrf2 protein level was reduced while p-NF-κB p65 protein level was enhanced in HRPs. Under this condition, CTRP3 elevation increased Nrf2 protein level while decreased NF-κB p65 protein level. As a consequence, after FOXO4 was overexpressed, the impacts of CTRP3 on Nrf2 and NF-κB p65 protein levels were both restored again.

## Conclusion

To be concluded, CTRP3 negatively modulated by FOXO4 could enhance the viability while attenuate the apoptosis and oxidative damage of HRPs upon exposure to HG via mediating Nrf2/NF-κB pathway. All these findings implies that CTRP3 might exert protective effects in HG-induced HRPs damage, which might provide effective therapeutic strategies for DR from a brand-new perspective.

## Data Availability

The analyzed data sets generated during the present study are available from the corresponding author on reasonable request.

## Highlights

CTRP3 is activated by FOXO4 down-regulation in DR

CTRP3 inhibites HG-induced oxidative damage of HRPs in DR

CTRP3 can regulate Nrf2/NF-κB signaling pathway
